# Energy and energy policy: chemistry’s role

**DOI:** 10.1186/1752-153X-6-S1-I1

**Published:** 2012-04-23

**Authors:** Steve Berry

**Affiliations:** 1 Department of Chemistry and The James Franck Institute, University of Chicago, Chicago, IL, USA

## 

This supplement to the *Chemistry Central Journal* (CCJ) is meant to serve at least two functions. One, consistent with the long-term goal of this journal, is to bring to its audience the full span of topics and areas in which chemistry plays a key role, even if it is not the only key contributing subject. Obviously energy is very much such a subject; chemistry plays an integral role in the acquisition, generation, distribution and efficient use of energy. But its role is only fully meaningful when chemistry’s contributions are integrated with those of other sciences such as climatology, and, every bit as important, with economics, sociology and political decision-making. This brings us to the second function of this supplement. It consists of a set of six papers developed in a course at the University of Chicago in the Fall term of 2010, and then later revised a bit for CCJ. Each paper was the work of *an interdisciplinary team*, consisting of three to five students; each team had to include at least one student in a natural science and at least one in a social science such as economics or public policy. A primary goal of the course was educating the students, all fourth-year undergraduates or graduate students, in working together across interdisciplinary lines, learning to convey the relevant concepts of their own field to others in very different fields and to integrate the concepts from very different areas to come to a coherent analysis of their chosen topic.

Each team made a progress report to the class midway through the term, and then made a final presentation in one of the last sessions. Students were encouraged to ask questions and make critical comments about papers prepared by other teams.

Each team could choose a topic from a list of suggestions or request approval of a topic of their own invention. The papers selected for this supplement were ones deemed particularly relevant by the two people giving the course, Professor George Tolley, an economist, and myself. Both of us have worked for many years in various aspects of energy, and a good fraction of that work was done together. It might be relevant here, to give a sense of the environment in which the papers were written, to point out that Professor Tolley and I agreed on some things and openly disagreed and argued in class about some other things. So these papers were written in a context in which questioning, challenging and testing ideas was pervasive. And the course continues!

## Competing interests

The author has no competing interests in this material.

